# Primary Ovarian Poorly Differentiated Adenocarcinoma with Signet-Ring Cells: A Case Report and Literature Review

**DOI:** 10.3390/jcm15010144

**Published:** 2025-12-24

**Authors:** Yu-Jin Koo, Min Hye Jang, Dae-Hyung Lee

**Affiliations:** 1 Department of Obstetrics and Gynecology, Yeungnam University Medical Center, 170, Hyeonchung-ro, Nam-gu, Daegu 42415, Republic of Korea; 2 Department of Pathology, Yeungnam University Medical Center, 170, Hyeonchung-ro, Nam-gu, Daegu 42415, Republic of Korea

**Keywords:** ovarian neoplasm, signet ring cell carcinoma, krukenberg tumor, mucinous adenocarcinoma

## Abstract

**Background:** Primary ovarian signet-ring cell carcinoma (POSRCC) is exceedingly rare. Most previously reported cases have involved primary ovarian mucinous neoplasms containing signet-ring cells. **Methods:** We report a unique case of advanced primary ovarian adenocarcinoma with a signet-ring cell component, accompanied by features inconsistent with typical mucinous morphology, which distinguished it from most previously documented cases. A review of the literature is also provided. **Results:** A 57-year-old woman presented with abdominal pain. Imaging revealed a 10 cm pelvic mass. Surgical exploration and pathological examination revealed poorly differentiated adenocarcinoma with focal signet-ring cell features in both ovaries. Extensive preoperative and postoperative evaluation revealed no evidence of an alternative primary tumor. The tumor did not meet diagnostic criteria for mucinous carcinoma. Therefore, the final diagnosis was primary ovarian poorly differentiated adenocarcinoma with a focal signet-ring cell component, FIGO stage IIIC. The patient received six cycles of adjuvant paclitaxel and carboplatin, followed by four cycles of single-agent bevacizumab for maintenance therapy. Despite treatment, disease recurred eight months after surgery, and the patient died of disease progression 18 months postoperatively. **Conclusions:** This case highlights the aggressive behavior of non-mucinous POSRCC and underscores the diagnostic challenge in distinguishing primary from metastatic ovarian signet-ring cell carcinoma. Awareness of this rare entity is crucial for accurate diagnosis and appropriate management.

## 1. Introduction

Signet-ring cell carcinoma (SRCC) is a distinct subtype of adenocarcinoma characterized by abundant intracytoplasmic mucin accumulation within individual tumor cells or small clusters of cells [1]. In the ovary, signet-ring cells most commonly occur in metastatic mucinous carcinomas, classically referred to as Krukenberg tumors. A Krukenberg tumor typically represents metastatic disease originating from primary malignancies of the stomach, pancreas, biliary tract, appendix, or colon [2]. Clinically, the term encompasses any ovarian metastatic carcinoma arising from an extragonadal primary site, although the definitive diagnosis relies on the World Health Organization (WHO) pathological criteria [3].

On rare occasions, SRCCs can arise as primary ovarian tumors. To date, only a limited number of primary ovarian SRCCs (POSRCCs) have been documented. Most previously reported cases describe primary ovarian mucinous neoplasms containing focal or extensive signet-ring cell features rather than neoplasms in which signet-ring cells constitute the predominant component of the tumor [4]. Moreover, mucin-laden signet-ring–like cells can also be encountered in other ovarian tumors, including clear cell carcinoma [5] and the small cell carcinoma of hypercalcemic type (SCCHT) [6].

Here, we present a case of primary advanced ovarian adenocarcinoma with a signet-ring cell component admixed with a background inconsistent with the typical morphology of mucinous tumors, thereby differing from most previously reported cases. We also provide a review of the relevant literature.

## 2. Case Report

### 2.1. Clinical Presentation

**Figure 1:**
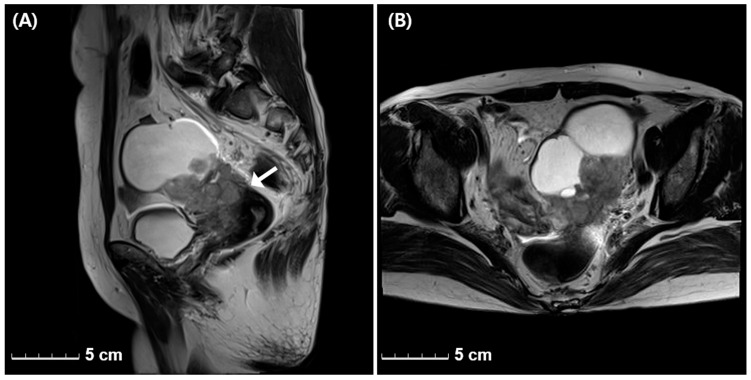
Sagittal (**A**) and axial (**B**) T2-weighted pelvic MRI images demonstrate an approximately 10 cm lobulated heterogeneous mass in the left adnexa that is abutting the superior and left lateral aspects of the uterus (arrow).

A 57-year-old postmenopausal woman, gravida 2 para 2, was referred to our outpatient clinic with lower abdominal pain lasting for 10 days. She had no significant past medical or surgical history other than hypertension, and no family history of cancer. Her body mass index was 21.3 kg/m^2^. Transvaginal ultrasonography revealed a 10 cm heterogeneous mass in the left adnexa. Laboratory findings for serum and urine were within normal limits. Her serum CA-125 level was 6.8 U/mL (reference range, <35 U/mL), and the Risk of Ovarian Malignancy Algorithm (ROMA) score was 11.8%, indicating a low risk of ovarian malignancy. Magnetic resonance imaging (MRI) showed a 10 cm lobulated mass with both cystic and solid components in the left adnexa, findings suspicious for metastasis to the left pelvic lymph nodes, along with left hydronephrosis and hydroureter (Figure 1).

**Figure 2:**
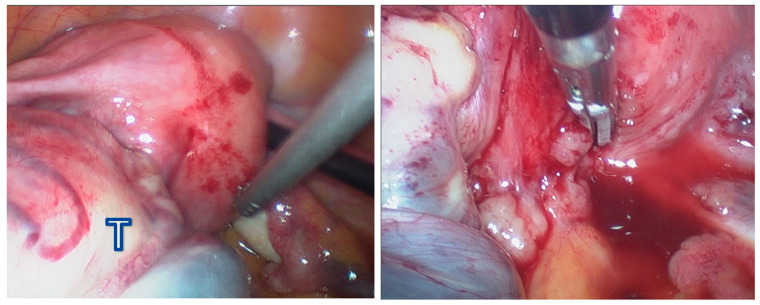
Laparoscopic findings demonstrate a 10 cm tumor on the left ovary (T), which is attached to the rectum.

Based on a presumptive diagnosis of ovarian cancer, exploratory laparoscopic surgery was performed. A 10 cm left ovarian tumor adherent to the uterus and the left ureter was identified. The right ovary appeared grossly normal, and no ascites was observed (Figure 2). Both pelvic and para-aortic lymph nodes were enlarged; however, no tumor implants were found in the omentum, abdominal peritoneum, or subdiaphragmatic area. The procedure was converted to an open laparotomy, and total abdominal hysterectomy, bilateral salpingo-oophorectomy, bilateral pelvic and para-aortic lymphadenectomy, appendectomy, and supracolic omentectomy were performed. Due to dense adhesion of the ovarian tumor to the left ureter and rectum, segmental resection followed by end-to-end anastomosis of both the left ureter and the rectum was performed by the departments of urology and colorectal surgery. At the conclusion of the surgery, no gross residual tumor was noted. The tumor was pathologically diagnosed as a poorly differentiated adenocarcinoma with signet-ring cell features.

A comprehensive postoperative workup was conducted to identify a possible primary site outside the ovaries. This included gastroscopy, colonoscopy, breast mammography, breast ultrasonography, hepatobiliary ultrasonography, abdominopelvic computed tomography (CT), and positron emission tomography (PET)-CT from the skull base to the upper thigh. However, no evidence of malignancy was detected elsewhere. Serum tumor markers for gastrointestinal malignancies were not assessed preoperatively but were measured on postoperative day 8, with the following results: CA 19-9, 15.4 U/mL (reference range, 0–27 U/mL), and CEA, 4.27 ng/mL (reference range, 0–5 ng/mL). A final diagnosis of primary ovarian adenocarcinoma with a signet-ring cell component, classified as stage IIIC according to the International Federation of Gynecology and Obstetrics (FIGO), was established.

The patient subsequently received six cycles of adjuvant chemotherapy with paclitaxel and carboplatin, followed by four cycles of single-agent bevacizumab as maintenance therapy. After four cycles of bevacizumab, PET-CT revealed new metastatic lesions in the lungs and multiple lymph nodes. The progression-free survival (PFS) following surgery was therefore eight months. The patient then underwent palliative chemotherapy, consisting of three cycles of belotecan combined with cisplatin, followed by three cycles of single-agent pegylated liposomal doxorubicin. Despite these treatments, the disease continued to progress, and the patient died of the disease progression 18 months after the initial surgery.

### 2.2. Pathologic Findings

**Figure 3:**
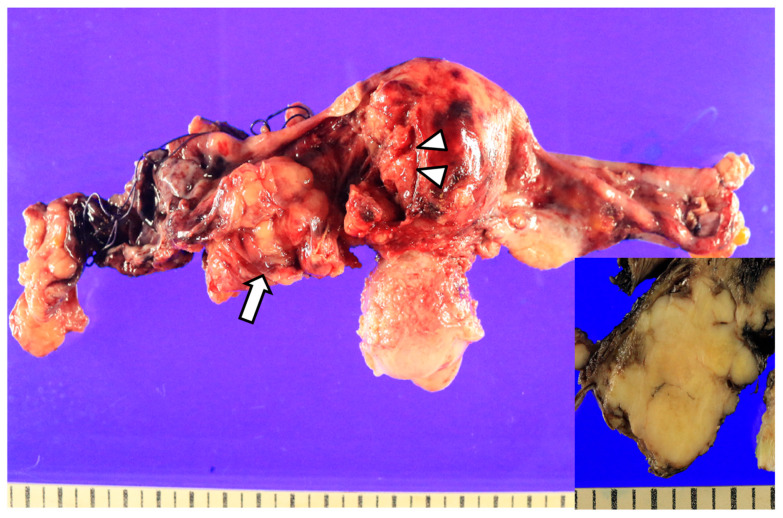
Posterior view of the gross specimen of the uterus and bilateral adnexa. The left ovary was replaced by a solid mass (white arrow), which directly invaded the uterine corpus (white arrowhead). The cut surface is homogeneously cream-colored (inset).

Macroscopically, the left ovary was entirely replaced by a solid mass measuring approximately 5.0 × 3.5 × 2.7 cm (Figure 3). The mass directly invaded the left wall of the uterine corpus, extending into the superficial myometrium over an area of approximately 3.6 × 1.3 cm. The tumor also infiltrated the deep muscular layer of the rectal wall and the periureteral soft tissue on the left side, without direct invasion of the ureteral wall. The right ovary had an intact capsule grossly. However, on the cut surface, a 0.7 × 0.5 cm solid nodule was identified, which was microscopically confirmed as metastatic carcinoma from the contralateral ovary. There was no tumor involvement of the cervix, endometrium, bilateral fallopian tubes, appendix, omentum, bladder peritoneum, or the vaginal vault excision specimen obtained after hysterectomy. A total of 23 lymph nodes were retrieved, including 4 right pelvic, 6 left pelvic, 6 right para-aortic, and 7 left para-aortic lymph nodes. Metastatic carcinoma was identified in one left pelvic and two left para-aortic lymph nodes. Peritoneal washing cytology also revealed metastatic carcinoma cells.

**Figure 4:**
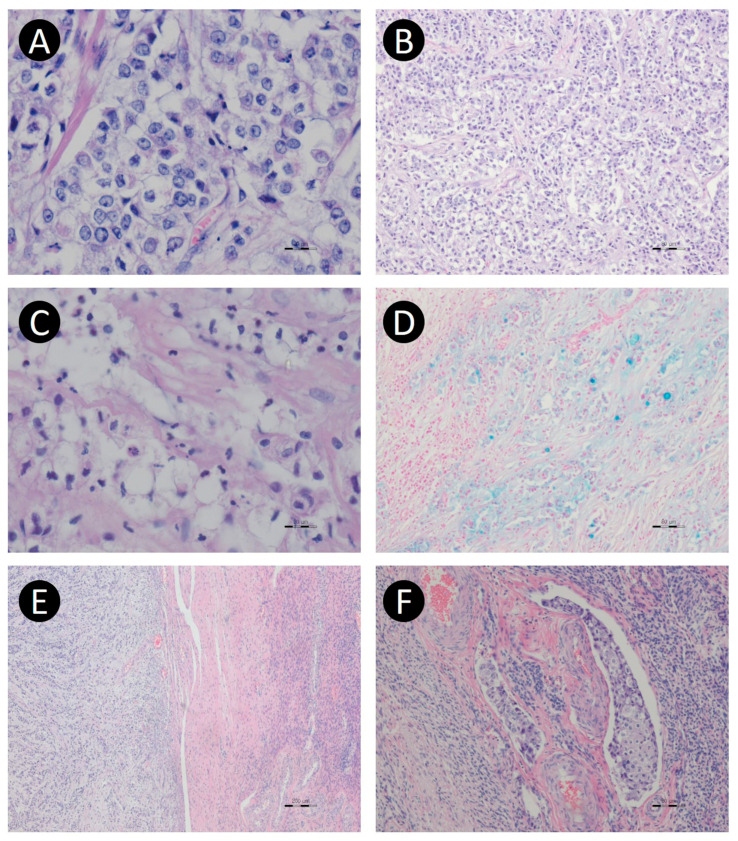
Microscopic images of ovarian carcinoma with a signet-ring cell component. (**A**) Most of the tumor was composed of large, highly pleomorphic polygonal tumor cells with vesicular nuclei and a prominent single nucleolus (×200). (**B**) Tumor cells showed a solid growth pattern or partly nested growth pattern. These major features resemble dysgerminoma or undifferentiated carcinoma. (**C**) However, some tumor cells had abundant clear or bubbly cytoplasm resembling signet-ring cells. (**D**) These tumor cells contained cytoplasmic mucin, which showed cytoplasmic blue staining with Alcian blue. (**E**) Metastatic tumor in the right ovary showed the same histologic features as the contralateral ovarian tumor. (**F**) Lymphovascular invasion was also frequently observed. [Original magnification: (**A**,**C**,**D**) ×200; (**B**,**E**,**F**) ×40].

Microscopically, the majority of the tumor was composed of solid sheets of monomorphic, round or polygonal tumor cells (Figure 4A,B). This component showed no specific differentiation, resembling undifferentiated carcinoma, and was frequently associated with geographic necrosis and a high mitotic index. A minor component of the tumor consisted of large, round, signet-ring cells containing abundant intracytoplasmic mucin (Figure 4C,D). The metastatic lesions, including that in the contralateral ovary, demonstrated the same histologic features (Figure 4E). Perineural invasion was absent; however, lymphovascular invasion was frequently observed in both the primary and metastatic tumors (Figure 4F). Tumor deposits on the ovarian surface with associated stromal infiltration were also identified. Both the vaginal and rectal resection margins were negative, whereas the left parametrial resection margin was involved by the tumor.

**Figure 5:**
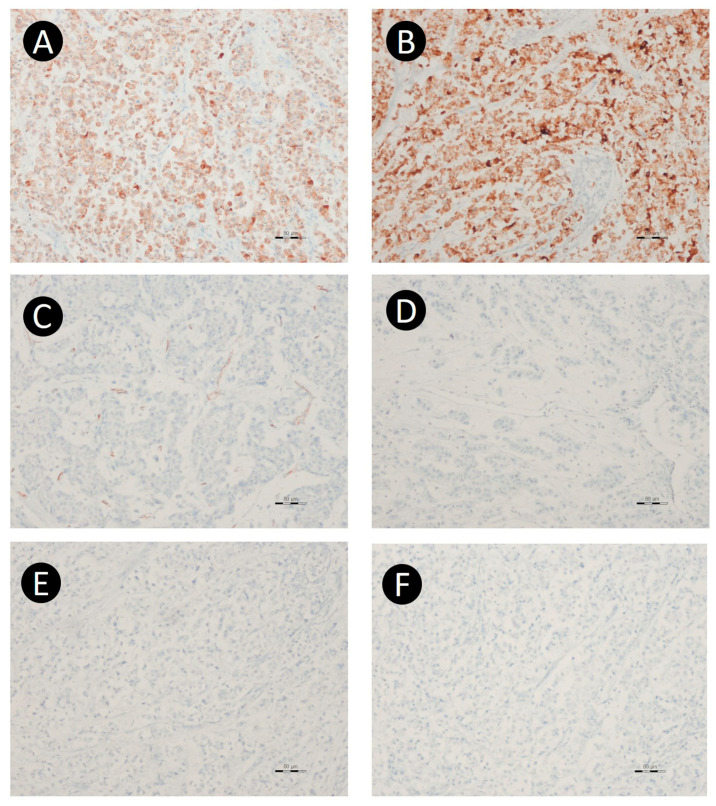
Representative immunohistochemistry of the ovarian tumor. The tumor cells expressed CK7 (**A**) and EMA (**B**), and were negative for WT-1 (**C**), CK20 (**D**), PAX8 (**E**), and GATA3 (**F**). [Original magnification: ×100].

Immunohistochemically, the tumor cells expressed CK (AE1/AE3), EMA, and CK7, but were negative for p53, CK20, CDX2, inhibin-α, WT-1, calretinin, ER, chromogranin, synaptophysin, CD56, PAX8, and GATA3 (Figure 5). Signet-ring cells demonstrated intracytoplasmic mucin that was positive for both periodic acid–Schiff (PAS) and alcian blue stains, confirming their identity as true signet-ring cells. Based on the immunohistochemical and special staining results, the tumor was classified as an adenocarcinoma with signet-ring cell features. To further characterize the tumor, next-generation sequencing (NGS) was performed on formalin-fixed, paraffin-embedded (FFPE) tumor tissue, targeting a panel of 143 cancer-related genes, including WT1, TP53, ERBB2, KRAS, GNAS, HRAS, BRCA1, BRCA2, EGFR, BRAF, KIT, PDGFRA, MYC, MYCN, and NRAS. No pathogenic variants were identified within the limitations of the targeted NGS panel.

## 3. Discussion

In the present study, we conducted a comprehensive review of cases reported up to October 2025. A systematic search was performed in PubMed using the search term “signet-ring cell”. Only full-text articles published in English were included to ensure a thorough evaluation.

### 3.1. Pathological Differences Between Metastatic and Primary Ovarian SRCC

Distinguishing POSRCC from metastatic disease is often challenging. Accurate differentiation between primary and secondary ovarian SRCC is clinically crucial, as therapeutic strategies differ substantially. Several clinicopathologic characteristics favor a metastatic origin, including bilateral ovarian involvement, small tumor size, multinodular growth, marked histologic heterogeneity, lymphovascular invasion—particularly at the ovarian hilum—tumor deposits on the ovarian surface, stromal infiltration, tumor cells floating within mucin pools, and evidence of extraovarian spread [7,8,9].

Conversely, features more suggestive of a primary ovarian neoplasm include unilateral involvement, large tumor size, early-stage presentation, a background of mucinous adenoma or adenofibroma, and the presence of endometriosis [2].

However, these clinicopathologic characteristics are not absolute diagnostic criteria for distinguishing primary from metastatic ovarian tumors, and overlap between the two entities has been reported. Consistent with this observation, bilateral ovarian involvement was observed in 51 of 172 cases of POSRCC (29.7%) in one study [1]. Furthermore, in a literature review of 11 cases of POSRCC, tumor deposits on the ovarian surface and lymphovascular invasion were identified in 2 (18.1%) and 1 (9.1%) cases, respectively [10]. In addition, in a series of non-mucinous POSRCCs, only one of nine reported cases presented at FIGO stage I, whereas the remaining cases were diagnosed at advanced stages (FIGO III–IV) [11].

In the present case, the tumor was interpreted as a primary ovarian neoplasm despite the presence of several clinicopathologic features commonly associated with metastatic SRCC. These included bilaterality, multinodular growth, lymphovascular invasion, ovarian surface involvement, stromal infiltration, and extraovarian spread; however, other features more characteristic of metastatic disease—such as small tumor size, marked histologic heterogeneity, and tumor cells floating within mucin pools—were absent. Notably, similar findings may also be encountered in advanced stage primary ovarian carcinomas, and a comprehensive clinical evaluation failed to identify an extraovarian primary site. Taken together, these observations suggest that the clinicopathologic features observed in this case reflect the intrinsic biological behavior of primary ovarian non-mucinous adenocarcinoma rather than metastatic disease.

### 3.2. Immunohistochemical Markers for Distinguishing Primary Ovarian Tumors from Metastases of Lower Intestinal Tract Origin

Previous reports have described cases of Krukenberg tumors in which the primary site remained uncertain, likely because the originating malignancy was too small or clinically silent to be detected despite a thorough diagnostic workup [12]. Immunohistochemical staining can aid in differentiating primary ovarian tumors from metastatic carcinomas of gastrointestinal origin.

CK7/CK20 immunoprofiles are well established as valuable tools for distinguishing primary ovarian mucinous tumors from metastases originating in the lower intestinal tract (appendix, colorectum). Metastatic tumors of lower intestinal origin typically show diffuse CK20 expression with absent or minimal CK7 expression, whereas primary ovarian mucinous tumors more often demonstrate diffuse CK7 positivity with variable CK20 staining—commonly present but usually patchy rather than diffuse [13].

Additionally, primary ovarian carcinomas may exhibit variable positivity for keratin 20, CEA, and CDX2 [10]. However, the diagnostic utility of immunohistochemistry becomes limited when distinguishing between primary and secondary ovarian mucinous carcinomas containing signet-ring cells, as these tumors frequently share overlapping immunophenotypic profiles.

PAX8, a marker of Müllerian origin expressed in tissues including the ovary, fallopian tube, and endometrium, is positive in most endometrioid, serous, and clear cell ovarian carcinomas, occasionally positive in mucinous ovarian carcinomas, and typically negative in Krukenberg tumors [14]. This makes PAX8 a useful marker for distinguishing primary epithelial ovarian carcinoma from metastatic ovarian carcinoma. However, since the majority of POSRCC cases are admixed with mucinous adenocarcinoma, the diagnostic value of PAX8 for differentiating POSRCC from metastatic ovarian carcinoma is limited. In a previous report of mucinous POSRCC, mucinous areas were focally positive for PAX8, whereas signet-ring cells were negative [10]. However, other authors have reported that malignant mucinous elements, including signet-ring cells, were negative for PAX8 [9,15].

### 3.3. Incidence of Ovarian SRCCs

Krukenberg tumors are rare, accounting for approximately 2% of all ovarian cancers [12]. POSRCC, however, is even rarer. In most previously published studies, signet-ring cells contained mucin and were typically admixed with other epithelial cell types within the tumor. The histopathology of ovarian epithelial neoplasms is complex and heterogeneous, and most reported cases of POSRCC have been observed in association with mucinous adenocarcinoma.

In 2021, Wang et al. reported POSRCC data from 1975 to 2016 using the most recent version of the U.S. SEER database, representing the largest POSRCC cohort published to date [1]. They identified 198 patients with POSRCC, of whom 172 had complete information and were included in the survival analysis. However, the study did not provide details regarding admixed epithelial components or the presence of an accompanying or underlying tumor. As the authors acknowledged, potential selection bias exists because POSRCC cases were identified solely through ICD-O-3 histology codes, and the wide study period raises concerns regarding variability in diagnostic standards and clinicopathologic assessment over time.

The second largest study on POSRCC was published in 1968, in which Joshi conducted an extensive review of 18 cases designated as “Primary Krukenberg Tumors of the Ovary” [16]. This study likewise did not describe the specific histologic subtype of the ovarian carcinomas, and given the older publication date, the diagnostic workup for determining tumor origin was likely less comprehensive than that used in current practice.

Additionally, we identified two other reports describing cases of POSRCC in which the epithelial cell type of the carcinoma was not clearly documented [12,17].

### 3.4. POSRCC in Mucinous Adenocarcinoma

In 2024, Gündoğdu et al. published a comprehensive literature review of POSRCC associated with mucinous adenocarcinoma, identifying a total of 11 cases, including one from their own institution [10].

However, additional cases of POSRCC have been documented in association with mucinous adenocarcinoma admixed with other benign or malignant histologic components. Vang et al. reported three cases of ovarian mucinous carcinomas containing an admixed SRCC component arising within mature cystic teratomas [13]. Indeed, mature cystic teratoma is one of the most commonly observed benign conditions associated with mucinous POSRCC. In recent studies, several researchers have indicated that a subset of intestinal-type mucinous tumors arising in association with mature teratomas may closely resemble lower gastrointestinal tract-type mucinous neoplasms, including occasional signet-ring cell morphology [13,18].

Additional reports include a unique case in which both a signet-ring cell mucinous adenocarcinoma and a pulmonary-type adenocarcinoma arose within a dermoid cyst [15]. Finally, Khadang et al. described the first reported case of a primary ovarian mixed malignant Brenner–mucinous tumor containing signet-ring cells [4].

### 3.5. POSRCC in Non-Mucinous Carcinoma

To date, at least 17 cases of POSRCC admixed with non-mucinous ovarian carcinomas have been reported. One of the largest series was published in 2001 by Che et al., who described the presence of signet-ring cells in epithelial ovarian carcinomas, including 15 cases of non-mucinous POSRCC (13 serous, one endometrioid, and one with mixed serous and endometrioid differentiation) [11]. They identified ovarian carcinomas characterized by a predominant microcystic pattern with signet-ring cells, accompanied by serous or endometrioid differentiation. This represented the first description of a microcystic pattern in POSRCC.

In 2002, another group reported the first case of an ovarian clear cell carcinoma in which cytologic specimens obtained by fine-needle aspiration of an ovarian mass showed predominantly signet-ring cells [19]. The diagnosis of POSRCC with clear cell carcinoma was subsequently confirmed on postoperative histologic examination. More recently, in 2013, a case of POSRCC associated with SCCHT was reported in a 35-year-old patient [20].

In the present case, the tumor did not fulfill the diagnostic criteria for mucinous tumors, which are characterized by glands and cysts lined mainly by columnar and cuboidal cells containing abundant intracytoplasmic mucin in most areas. Therefore, the case was diagnosed as a poorly differentiated adenocarcinoma with signet-ring cells of the ovary and it was classified as ovarian adenocarcinoma, not otherwise specified (NOS), according to the 2020 WHO Classification of Female Genital Tumors (5th edition). Several entities were considered in the differential diagnosis, including mesonephric-like adenocarcinoma; however, the absence of characteristic morphologic features and the lack of PAX8 and GATA3 expression did not support this diagnosis.

Unlike previously reported cases of POSRCC associated with non-mucinous adenocarcinoma, the present case was dominated by solid sheets of undifferentiated-like tumor cells with only a focal signet-ring cell component. Notably, no microcystic histologic pattern of the signet-ring cell component was identified. Accordingly, this case represents a previously unreported variant of POSRCC characterized by a predominant undifferentiated component admixed with a minor signet-ring cell component. In addition, the study by Che et al. did not provide a detailed immunohistochemical profile, reporting only CK7 positivity and CK20 negativity in the POSRCC [11]. In the present case, we performed a more comprehensive immunohistochemical evaluation, together with NGS analysis, and similarly demonstrated CK7 positivity and CK20 negativity.

### 3.6. Clinicopathologic Characteristics of POSRCC

Owing to the rarity of POSRCC, its clinical characteristics remain poorly defined. In a literature review of 11 cases of POSRCC admixed with mucinous adenocarcinoma, the patients’ ages ranged from 24 to 78 years, with a mean age of 47.9 years, and tumor sizes ranged from 9 to 30 cm, with a mean size of 20.1 cm [10]. In 3 of the 11 cases (27.3%), signet-ring cells constituted the predominant component, whereas in the remaining 8 cases (72.7%), signet-ring cell foci were present only focally, ranging in size from 0.1 to 5 cm. Most cases were diagnosed at FIGO stage I, although two cases were diagnosed at stage III.

In contrast, in a series of 15 cases of POSRCC admixed with microcystic-pattern non-mucinous adenocarcinomas, signet-ring cells were diffusely present in 9 tumors (60%) and focally present in the remaining 6 tumors (40%) [11]. Among these nine patients with available clinical information, ages ranged from 31 to 78 years, with a mean of 58 years. Most cases were diagnosed at FIGO stage III or IV, with only one case at stage I.

We speculate that this apparent difference in tumor stage may reflect differences in the underlying histology—mucinous versus non-mucinous—or the distribution of signet-ring cells, whether focal or diffuse.

### 3.7. Treatment of Ovarian SRCCs

In general, POSRCC is managed with surgery followed by adjuvant systemic chemotherapy. Although the optimal surgical approach for POSRCC has not been clearly established, complete cytoreductive surgery with no residual tumor is considered essential, similar to the management of conventional epithelial ovarian carcinoma.

In a 2024 literature review, three of the 11 patients received adjuvant paclitaxel/carboplatin chemotherapy [10]. Among these patients, only one experienced disease recurrence two years after diagnosis, and one patient died from pulmonary complications following treatment. Based on these follow-up data, the authors suggested that POSRCC may not require additional therapy after surgery in early-stage tumors, whereas adjuvant chemotherapy could be considered for higher-stage disease.

Regarding chemotherapy regimens, most reported cases of POSRCC have been treated with paclitaxel/carboplatin therapy. Notably, a single case of stage IB POSRCC was successfully treated with S-1/cisplatin (CDDP) therapy, with no recurrence observed 27 months after the initial treatment [17].

### 3.8. Prognosis of Ovarian SRCCs

Krukenberg tumors are associated with a uniformly poor prognosis, given the lack of standardized chemotherapy or radiotherapy; reported median survival ranges from 7.7 to 19.0 months [12]. Similarly, outcomes of POSRCC are generally poor, although survival varies considerably across published reports. According to one report, the aggressive behavior of POSRCC may be related to its wide histologic variability and intrinsic biological aggressiveness [17]. The tumor is also infiltrative and often demonstrates early lymph node involvement, peritoneal dissemination, and hematogenous metastasis, features similar to those observed in metastatic ovarian carcinoma.

According to a SEER database study by Wang et al., the median overall survival of POSRCC, irrespective of epithelial histology, was 7 months (95% confidence interval [CI], 4.6–9.4 months), comparable to that of Krukenberg tumors [1]. Reported 1-, 3-, and 5-year overall survival rates were 35.5%, 15.3%, and 6%, respectively, reflecting the generally poor prognosis of POSRCC. The authors developed a prognostic nomogram and risk stratification model to predict patient outcomes, and multivariable analysis identified age at diagnosis, race, marital status, T stage (primary tumor size), and chemotherapy as independent predictors of survival. However, as previously mentioned, the study did not report details regarding admixed epithelial components, including mucinous or non-mucinous elements.

Che et al. similarly reported poor outcomes for POSRCC admixed with non-mucinous adenocarcinomas [11]. Most patients presented with advanced stage disease, and clinical follow-up ranged from 7 to 90 months (mean, 40 months). Among nine patients, seven (77.8%) either died of disease (four cases) or were alive with disease (three cases), whereas only two (22.2%) were alive with no evidence of disease, despite treatment with total abdominal hysterectomy, bilateral salpingo-oophorectomy, tumor debulking, and chemotherapy.

In contrast, a literature review of 11 cases of POSRCC admixed with mucinous adenocarcinoma reported that most tumors were stage I, and early-stage POSRCC was associated with a favorable prognosis even in the absence of adjuvant therapy [10].

In the present case, the tumor was diagnosed as a primary ovarian non-mucinous adenocarcinoma with focal signet-ring cells and presented at an advanced stage. Despite optimal cytoreductive surgery followed by chemotherapy and anti-angiogenic therapy, the clinical course progressed rapidly. These observations suggest that, in addition to tumor stage, the underlying histologic subtype may also play a critical role in the prognostication of POSRCC, similar to other epithelial ovarian cancers.

## 4. Conclusions

In summary, we report an extremely rare case of POSRCC presenting as a non-mucinous ovarian carcinoma with mucin-laden signet-ring cells. Although several characteristic features have been described to distinguish POSRCC from metastatic ovarian SRCCs, these features may not be evident in advanced stage POSRCC, which can complicate accurate diagnosis. A residual risk of an occult gastrointestinal or pancreatobiliary primary cannot be entirely excluded, as the originating malignancy may remain subclinical or below the threshold of clinical detection even after thorough evaluation. Therefore, differentiation from metastatic SRCCs requires comprehensive evaluation of clinical and pathological findings, including immunohistochemical staining.

In our literature review, most reported cases of POSRCC were admixed with mucinous adenocarcinomas, whereas only a few cases were described in non-mucinous carcinomas. However, underreporting of POSRCC and incomplete documentation of non-mucinous POSRCC cases may exist, given the wide and complex histologic spectrum of SRCC. Pathologists should be aware of these possibilities in primary ovarian tumors and their metastases to avoid misdiagnosis as metastatic disease from a non-ovarian neoplasm.

Although treatment outcomes of POSRCC vary widely across reports, cases that are not definitively early-stage may warrant aggressive management with surgery and chemotherapy due to rapid disease progression and poor prognosis. In particular, the specific admixed histologic subtype containing signet-ring cells appears to be critical for predicting survival outcomes. Pathologists should carefully assess the non-mucinous components in POSRCC, since the underlying histologic subtype, rather than the quantity or mere presence of signet-ring cells, appears to have a stronger correlation with poor prognosis.

## Data Availability

The original contributions presented in this study are included in this article. Further inquiries can be directed to the corresponding author.
